# Consecutive electrocardiographic changes during percutaneous coronary intervention for acute coronary syndrome with high-grade atrioventricular block: a case report

**DOI:** 10.1186/s12872-020-01392-6

**Published:** 2020-02-24

**Authors:** Hiroyuki Sueyoshi, Yuzo Akita, Yohei Oishi, Yu Mukai, Tomoko Hagino, Kotaro Yutaka, Yumie Matsui, Masahiro Yoshinaga, Masahiro Karakawa, Yasukiyo Mori

**Affiliations:** 1 Division of Cardiology, Osaka Saiseikai Izuo Hospital, 3-4-5 Kitamura, Taisho-ku, Osaka, 551-0032 Japan; 2 Division of Nephrology, Osaka Saiseikai Izuo Hospital, 3-4-5 Kitamura, Taisho-ku, Osaka, 551-0032 Japan

**Keywords:** Ischemic heart disease, Acute coronary syndrome, High-grade atrioventricular block, Complete atrioventricular block, Percutaneous coronary intervention

## Abstract

**Background:**

Acute coronary syndrome (ACS) with high-grade atrioventricular block (HAVB) still has a poor mortality risk, even in the current percutaneous coronary intervention (PCI) era. However, early PCI for ACS with HAVB is associated with improved in-hospital survival and a 6-month survival similar to that of ACS without HAVB.

**Case presentation:**

A 70-year-old man was admitted to our hospital for ACS with HAVB. ECG showed complete AV block, complete right bundle branch block (CRBBB), and left axis deviation. Cardiac enzymes were elevated. He underwent temporary pacemaker insertion and coronary angiography, which showed severe stenosis of the proximal right coronary artery (RCA), 99% stenosis of the distal RCA with Thrombolysis in Myocardial Infarction (TIMI) grade 2 flow, and total occlusion of the proximal left anterior descending artery (LAD). We performed primary PCI in both the RCA and LAD, which resulted in TIMI grade 3 flow in both. After PCI, the HAVB recovered to normal sinus rhythm with CRBBB; a normal QRS interval returned within three days. The patient was discharged from the hospital without complications.

**Conclusion:**

In this case of ACS with HAVB, early intensive coronary artery reperfusion resulted in long-term patient survival. The blood supply to the AV node and bilateral bundle branches is complex. Multivessel ischemia may compromise both primary and collateral blood flows to the AV node and septum, resulting in severe conduction impairment. Clinicians performing PCI should be aware of this anatomy and physiology.

## Background

The ECG changes and arrhythmia that complicate acute coronary syndrome (ACS) are associated with a disruption of the heart conduction system or the myocardial anatomic nervous system anatomy [1]. High-grade atrioventricular block (HAVB) can occur in patients with either anterior or inferior myocardal infarction [2] and has been reported to occur in 2.9% of ACS cases. Even in the current PCI era, HAVB is associated with poor mortality [3]. However, long-term survival is similar between HAVB patients who survive the initial hospitalization and patients without HAVB [3], therefore appropriate PCI for the ischemic coronary lesions is important. In the present case, the patient had multivessel lesions that affected the conduction system below the His bundle.

## Case presentation

A 70-year-old man was transported to our hospital via ambulance due to orthopnea. He reported general fatigue and dyspnea in the preceding five days and was taking medications for hypertension, dyslipidemia, and diabetes mellitus. There was no prior history of heart failure or ACS. His family history was unremarkable.

On arrival, blood pressure was 131/62 mmHg. Heart rate was remarkably low at 35 beats/min. No cardiac murmur was auscultated, however there were moist rales in the lower lungs bilaterally.

**Fig. 1:**
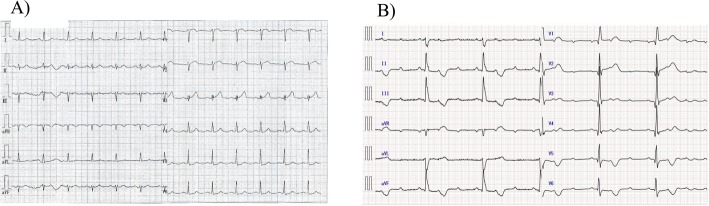
12-lead ECG. ECG sent from his practitioner shows normal sinus rhythm without conduction abnormality **a**. ECG on admission reveals complete atrioventricular block, complete right bundle branch block, and left axis deviation **b** ECG, electrocardiogram

**Fig. 2:**
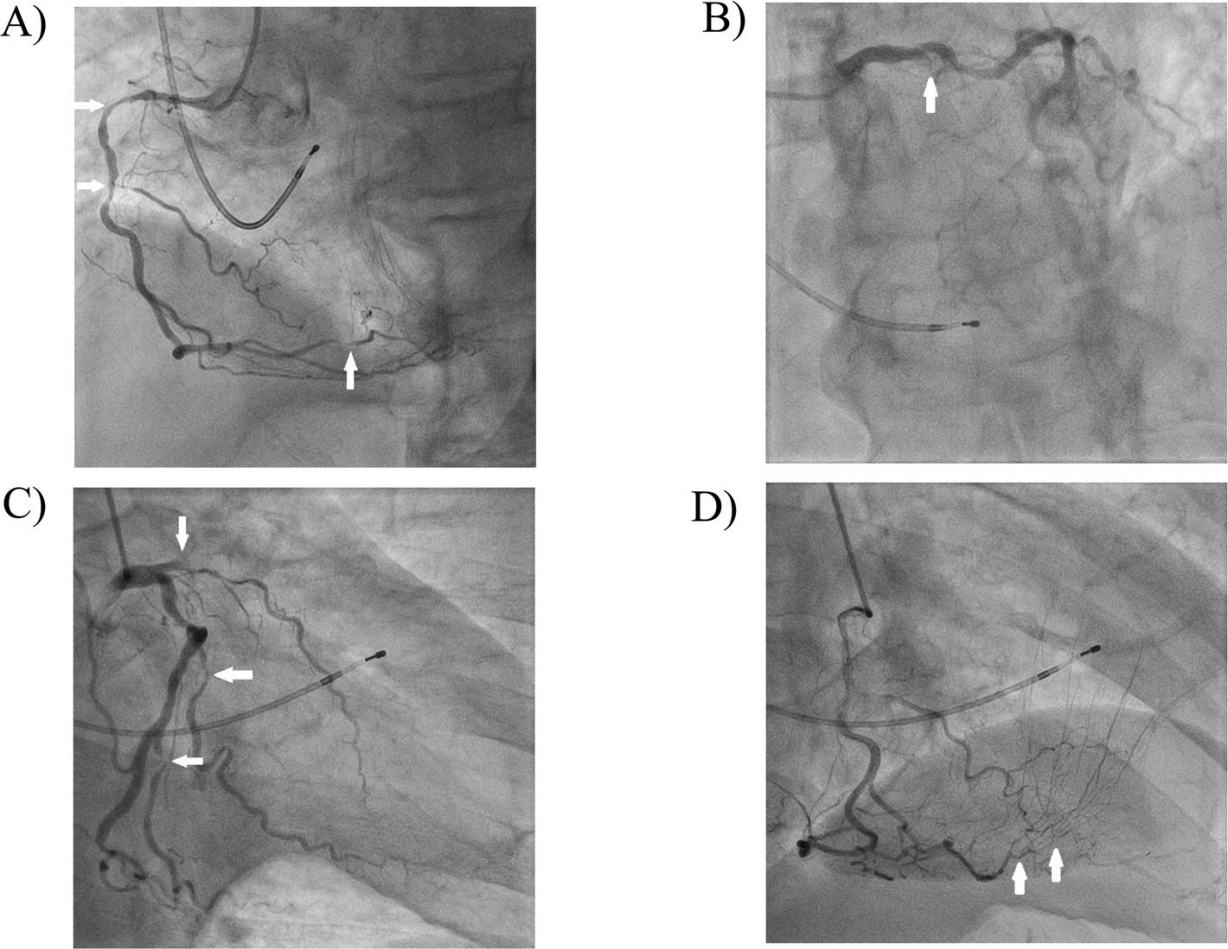
Emergency coronary angiography. There are multivessel lesions, including severe stenosis of the proximal, 99% distal RCA stenosis with TIMI grade 2 flow **a**, total occlusion of the proximal LAD) **b**, and severe stenosis of the distal circumflex artery at the obtuse marginal and posterolateral branches **c**. A collateral channel from the right ventricular branch of the RCA to the LAD is observed **d** LAD, left anterior descending artery; RCA, right coronary artery; TIMI, Thrombolysis in Myocardial Infarction

A previous ECG sent from his practitioner showed normal sinus rhythm without conduction abnormality (Fig. 1a). The admission ECG showed complete atrioventricular (AV) block, complete right bundle branch block (CRBBB), and left axis deviation (Fig. 1b). CK and troponin T enzymes were elevated (Table 1). Echocardiography showed a dilated left ventricle, severe hypokinesis of the anterior ventricle, and decreased ejection fraction (35%). There were no significant valvular abnormalities. We diagnosed him with ACS, HAVB, and congestive heart failure. A temporary pacemaker was immediately placed, followed by emergent coronary angiography (CAG). As Fig. 2 shows, there were multiple lesions, including severe stenosis of the proximal right coronary artery (RCA), 99% stenosis of the distal RCA with Thrombolysis in Myocardial Infarction (TIMI) grade 2 of the distal RCA flow, total occlusion of the proximal left anterior descending artery (LAD), and severe stenosis of the distal circumflex artery (Cx) at the obtuse marginal (OM) and posterolateral branches. A collateral channel from the right ventricular branch of the RCA to the LAD was also observed.

**Table 1:** Laboratory data on admission

	Reference Value		unit
White blood cell count	3900–9800	10,500	/μL
Creatine kinase	50–250	401	U/L
Creatine kinase MB	3–25	38	U/L
Aspartate aminotransferase	10–40	51	U/L
Alanine aminotransferase	5–45	16	U/L
Lactate dehydrogenase	115–245	237	U/L
Troponin T	0–49	597	ng/L
C-reactive protein	0–0.3	0.22	mg/dL
Glucose	70–109	507	mg/dL
Hemoglobin A1c	4.6–6.2	6.3	%
LDL-cholesterol	70–139	121	mg/dL

**Fig. 3:**
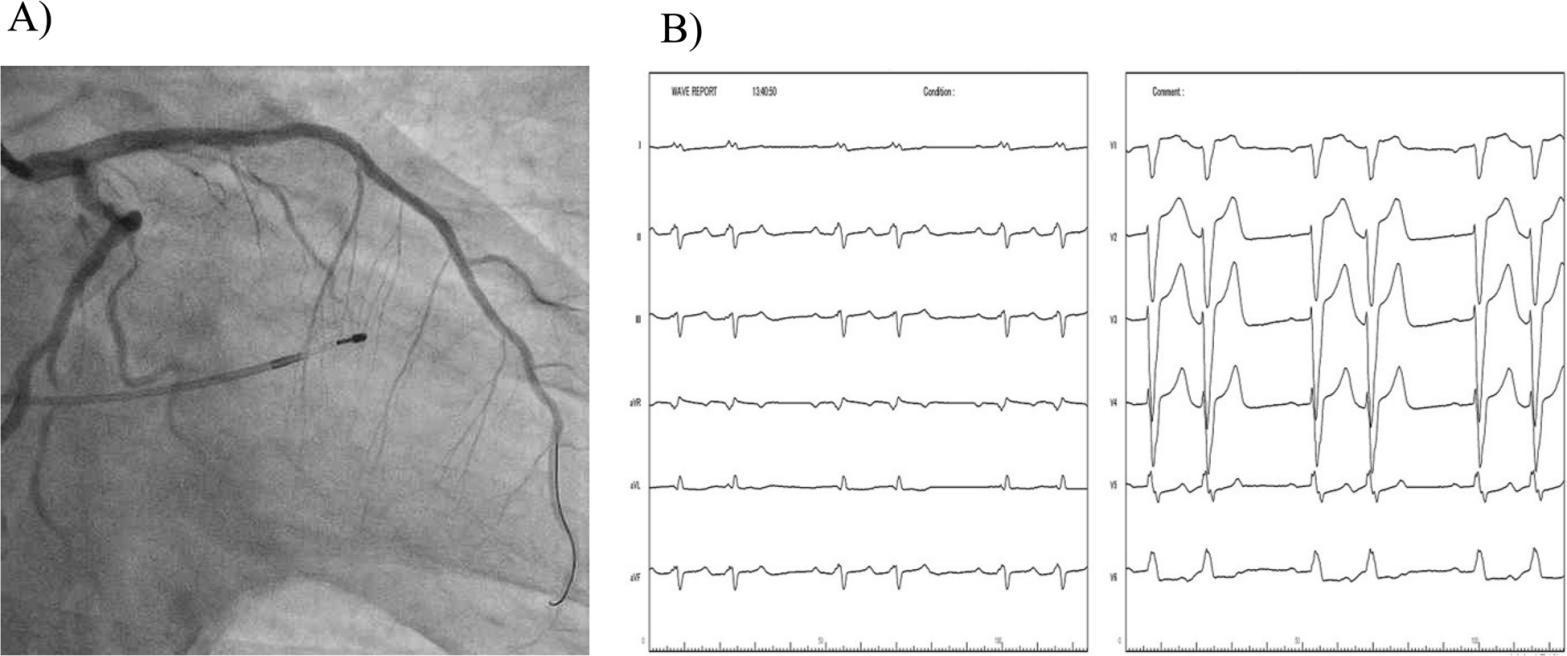
Coronary angiography and ECG after PCI to the LAD. Flow in the LAD improved to TIMI grade 3 **a**. The ECG showed Mobitz type 2 AV block with left bundle branch block **b** LAD, left anterior descending coronary artery; TIMI, Thrombolysis in Myocardial Infarction; ECG, electrocardiogram; AV, atrioventricular node

**Fig. 4:**
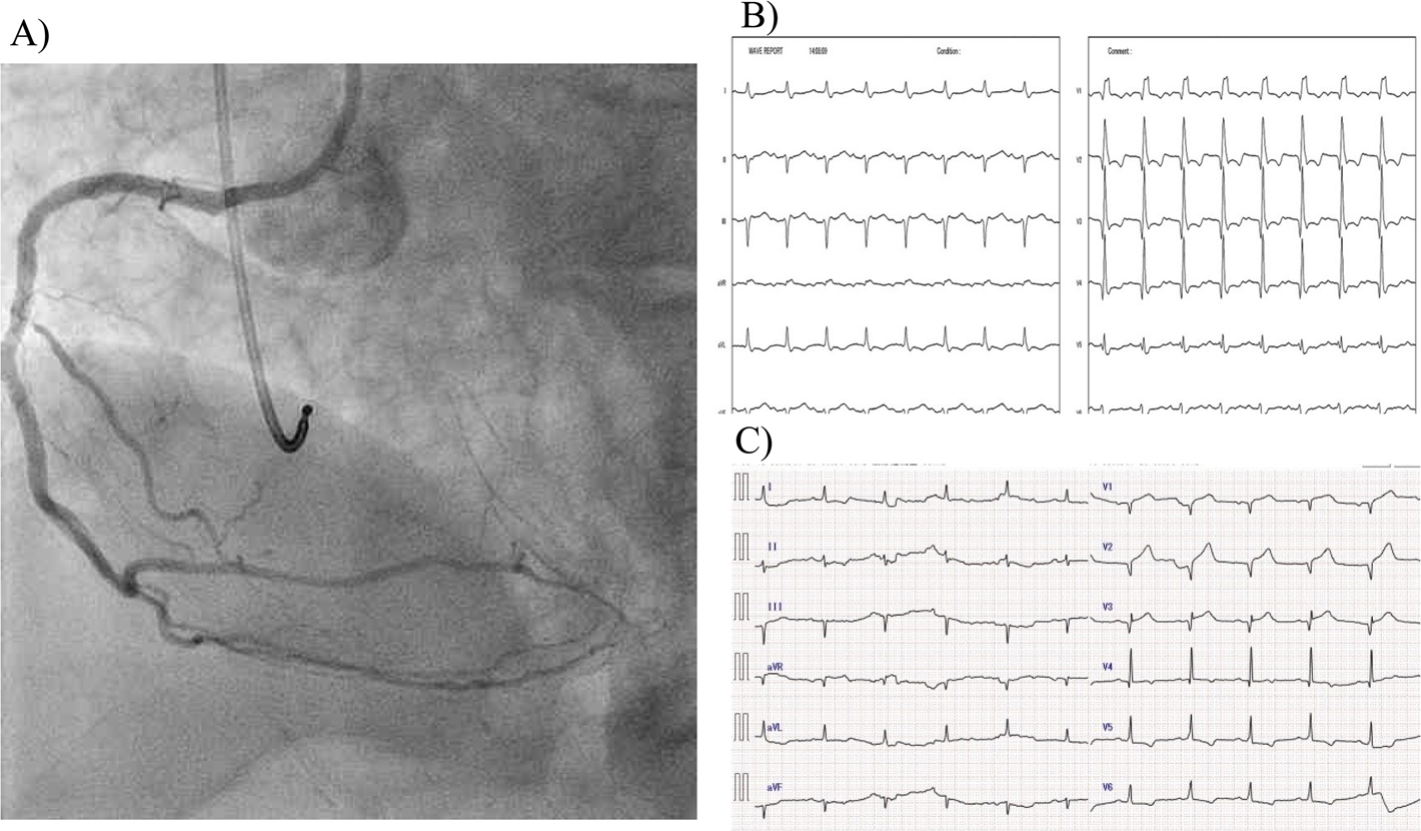
Coronary angiography and ECG after PCI to the RCA. Successful PCI to the RCA lesions is shown **a**. The ECG improved to normal sinus rhythm with CRBBB **b**. The prolonged QRS interval normalized within three days **c** PCI, percutaneous coronary intervention; RCA, right coronary artery; ECG, electrocardiogram; CRBBB, complete right bundle branch block

First, we performed primary PCI to the proximal LAD occlusion. Drug-eluting stents (DESs) were implanted in tandem (3.5 × 24 mm and 2.75 × 38 mm) from the left main trunk (LMT) to the mid portion of the LAD. Subsequently, kissing balloon dilatation was performed between the LMT–LAD and the Cx at the OM branch. Although intraaortic balloon pump insertion was necessary because of hypotension during the dilatation, the LAD improved to TIMI grade 3 flow (Fig. 3a). However, Mobitz type 2 AV block with left bundle branch block (LBBB) persisted (Fig. 3b); therefore, we performed additional PCI in the RCA. The distal and proximal RCA stenoses were treated with 2.0 mm and 2.5 mm balloon angioplasty, respectively. Because of slow flow phenomenon in the proximal RCA, intracoronary injection of nitroprusside and nicorandil was required to achieve TIMI grade 3 flow. Then, we implanted 3.0 × 24 mm and 2.5 × 38 mm DESs in tandem in the proximal RCA (Fig. 4a), after which the ECG improved to normal sinus rhythm with CRBBB (Fig. 4b). The prolonged QRS interval also normalized within three days after the primary PCI (Fig. 4c).

The temporary pacemaker was removed, and cardiac rehabilitation therapy was initiated. We additionally prescribed an ACE inhibitor, beta blocker, statin, and two types of antiplatelet agent with a proton-pump inhibitor. On the 28th hospital day, follow-up CAG and an electrophysiologic study were performed: the coronary stents were patent, and the AH and HV durations were normal at 97 msec and 45 msec, respectively. Although rehabilitation for his bilateral knee osteoarthritis was necessary, he was discharged from the hospital without cardiac symptoms on the 46th hospital day. During follow-up examination at our clinic, he remained free of complications for more than six months. Further revascularization of the Cx was not necessary.

## Discussion and conclusions

HAVB can occur in anterior and inferior acute myocardial infarction (AMI) [1–3]. Compared to patients with anterior AMI, patients with inferior AMI are more likely to develop complete AV block but are less likely to die prior to hospital discharge [1]. The usual location of the injured conduction system is proximal (His bundle) in inferior AMI and distal (bundle branches) in anterior AMI. HAVB in the setting of inferior AMI is usually transient for approximately 2–3 days and associated with 45–60 escape beats/ min with narrow QRS (< 0.12/s) interval. On the other hand, HAVB in the setting of anterior AMI usually presents with < 30 escape beats/min with wide QRS (> 0.12 s) interval and occasionally requires permanent pacemaker placement [4].

In the present case, ECG on admission showed 34 escape beats/min with wide QRS interval (0.17 s), suggesting injury in the distal AV conduction systems. Because multiple lesions of both left and right coronary arteries were observed, the lesion responsible for HAVB was difficult to identify immediately. The right bundle and the anterior fascicle of the left bundle are mainly supplied by the LAD. However, the posterior fascicle of the left bundle is supplied by the LAD, Cx, and RCA, which provides protection from ischemia [4]. In this patient, the ECG on admission showed complete AV block with CRBBB and left axis deviation, suggesting that the conduction system ischemia was below the His bundle, which is supplied by the both the LCA and RCA. Prior to the primary PCI, we hypothesized that the culprit lesion in this case was the RCA, which jeopardized the collateral channel to the LAD. Indeed, the anteroseptal wall showed edematous changes on cardiac magnetic resonance imaging, which was performed 8 days after the primary PCI (data not shown); these findings implied acute injury of the anteroseptal wall.

The consecutive ECG changes during PCI in this case can be explained anatomically. LAD reperfusion after primary PCI resulted in an ECG change to Mobitz type 2 AV block with LBBB, which was probably due to recovery of the right bundle. The additional RCA reperfusion improved the rhythm to normal sinus. This implied that the left posterior fascicle recovered blood supply from both the LCA and RCA.

The recent global registry of 59,229 patients with a broad spectrum of ACS showed that the incidence of HAVB (2.9%) was lower than previous reports [1, 3, 5]. Although the in-hospital mortality of HAVB in ACS did not decrease, the post-discharge mortality at six months was not significantly different between patients with and without HAVB (adjusted OR 1.06, 95% confidence interval (CI) 0.8–1.4). Increased in-hospital survival among patients with HAVB at presentation was associated with receipt of fibrinolysis and/or PCI within 12 h of hospitalization (adjusted OR 0.51, 95% CI 0.36–0.71) [3]. Therefore, aggressive care, including early PCI, may be necessary to improve outcome in patients with HAVB.

In this case of ACS with HAVB, careful coronary artery reperfusion for each lesion in the early phase resulted in long-term survival. Because the coronary artery blood supply to the distal His conduction system is complex, multivessel ischemia may compromise both primary and collateral blood flows to the AV node and septum, resulting in severe conduction impairment. Clinicians performing PCI should be aware of this anatomy and physiology.

## Data Availability

The datasets generated during and/or analyzed during the current study are available from the corresponding author on reasonable request.

## Abbreviations

ACS: Acute coronary syndrome
AMI: Acute myocardial infarction
CAG: Coronary angiography
CRBBB: Complete right bundle branch block
DES: Drug-eluting stents
HAVB: High-grade atrioventricular block
LAD: Left anterior descending
LBBB: Left bundle branch block
LMT: Left main trunk
OM: Obtuse marginal
RCA: Right coronary artery
TIMI: Thrombolysis in myocardial infarction
